# Types of devices used in ridge split procedure for alveolar bone expansion: A systematic review

**DOI:** 10.1371/journal.pone.0180342

**Published:** 2017-07-21

**Authors:** Nayansi Jha, Eun Ha Choi, Nagendra Kumar Kaushik, Jae Jun Ryu

**Affiliations:** 1 Department of Prosthodontics, Graduate School of Clinical Dentistry, Korea University Anam Hospital, Seoul, Republic of Korea; 2 Plasma Bioscience Research Centre and Department of Electrical & Biological Physics, Kwangwoon University, Seoul, Republic of Korea; Institute of Materials Science, GERMANY

## Abstract

The aim of this systematic review was to evaluate instrumentation procedures of the alveolar ridge expansion technique (ARST) with or without Guided Bone Regeneration (GBR) and to identify the most used instruments for successful outcome. An electronic as well as manual literature search was conducted in several databases including Medline, Embase, and Cochrane Central Register of Controlled Trials, for articles written in English up to September 2016. The question in focus was to identify the type of device for ridge expansion that is most frequently used and provides adequate bone expansion and implant success rate. To meet the inclusion criteria, the studies were analysed for the following parameters: prospective or retrospective studies, cohort or case studies/series, cases with 5 or more human subjects, type of device used for surgery, location of defect, and minimum follow up period. The frequency of osteotome usage in this study was approximately 65%, and on average, the implant success was 97%. The motorized expanders and ultrasonic surgery system are easier to use and cause less trauma to the bone compared to the traditional/conventional instruments like mallets and osteotomes. However, their cost is a limiting factor; hence, osteotomes remain a popular mode of instrumentation.

## Introduction

Earlier, ridge-widening techniques were used as a form of pre-prosthetic ridge plasty for providing support to partial/full dentures. With the introduction of root form implants and the concept of osseo-integration, the ridge plasty technique has once again become popular [1]. The concept for this novel technique was introduced by Tatum in 1986. Simion et al. [2] and Sciopini et al. [3] introduced the bone splitting technique using chisels for ridge expansion [4, 5]. A few literature reports depict different modifications of the ridge-split procedure (RSP) with or without inter-positional bone grafting in the edentulous maxilla and mandible [1].

If the alveolar bone width is 3mm or greater but less than 6mm, the alveolar ridge augmentation using a ridge splitting and bone expansion technique may be performed, for successful implant placement. At least 1mm of trabecular bone should be present between the cortical plates, when considering the minimum alveolar bone width for surgical purposes. This will allow the bone to spread adequately on either side of the ridge and maintain adequate blood supply. Several ridge split techniques have been developed in the past few decades, including split crest osteotomy, ridge expansion osteotomy, and various other modifications [4].

Ridge splitting is a technique-sensitive procedure that may be performed with many different instruments, ranging from chisel and mallet to scalpel blades, spatula, osteotomes, piezoelectric surgical systems, lasers, and ultra-fine fissure burs. The alveolar ridge expansion creates a self-space making defect [5] in the atrophied alveolar crests [2, 6]. When any instrument is used on the alveolar ridge (for ridge expansion), the mechanism involves inducing a greenstick fracture with lateral positioning of the buccal cortical plate. A longitudinal osteotomy takes places with the formation of a new implant bed [5]. Amongst the various instruments used for ridge expansion, osteotomes are the most popular ones. Gonzalez et al. [7] in their study, strongly advocated using the osteotomes to avoid unwanted fracture lines in the buccal or lingual cortical plates. Padmanabham et al. [8] showed that lesser resonance frequency was generated with an osteotome than with conventional techniques. There was more primary stability and lesser bone loss with gradual bone expansion, and heat generated due to instrumentation was minimal [9] with osteotomes.

One of the traditionally used devices is the chisel and (hand) mallet. Nowadays, electrical or magnetic mallet has been introduced, which is used in combination with the osteotomes. The osteotome is attached to the hand-piece (mallet), which transmits shock waves to the tip of the instrument, thereby creating longitudinal movements on the bone surface [10]. Crespi et al. [10] advocated the use of magnetic mallet instead of hand mallet as it provided more comfort and stability to the operator.

The modern devices used for ridge expansion include motorized ridge expanders, expansion crest device, and piezoelectric device used for ultrasonic bone surgery. They are non-cutting drills that can facilitate width expansion of atrophic ridges without using a surgical mallet; they can also be used as condensers of trabecular bone [11].

Usage of expansion crest device for ridge expansion can also be considered one of the alternatives to conventional techniques. The main advantage of using this device is that it allows distribution of expansion forces, which helps in preventing bone removal from the buccal cortex, and adequate site preparation can be achieved. The device has been used most successfully in areas that have cancellous bone in the edentulous ridge [12].

The piezoelectric surgery systems are the newest crest expansion devices in dentistry. They work on the principle of piezoelectric effect, which was discovered in the 1880s [13]. In comparison to other alternatives for bone cutting procedures, the ultrasonic or the piezoelectric device has been found to be the most effective. With this device, selective cutting of the bone without affecting the soft tissue (nerves and blood vessels) may be carried out [14]; further, an oscillating tip with an irrigating fluid provides a cleaner working area and greater visibility (cavitation effect) at the surgical site [15] without causing bone heating (compared to conventional devices).

According to our knowledge, until now no systematic review has focused on evaluating the instrumentation techniques for ridge expansion. Therefore, this systematic review aimed to evaluate instrumentation procedures of alveolar ridge expansion techniques with or without GBR as well as their effect on survival rates of dental implants.

## Materials and methods

For the following review, we used Cochrane Collaboration [16] and Preferred Reporting Items of Systematic Reviews and Meta-analysis [17] to prevent any risk of bias.

### Question in focus

According to the PICO (problem, intervention, comparison, and outcome) model, we decided to address the following question; ‘What type of device for ridge expansion is most frequently used and provides adequate bone expansion and implant success rate?’

### Information source and search strategy

A scoping review was performed according to PRISMA statement [17] for systematic reviews (see S2 Appendix) for which an electronic and a manual literature search were conducted using several databases including Medline, Embase, and Cochrane Central Register of Controlled Trials, for articles written in English from inception up to September 2016. In the electronic search, the search string comprised a combination of key words (i.e., medical subject headings, MeSH) and free text terms. The linkage was conducted using Boolean operators (OR, AND). The following search strategy was applied: (Alveolar Bone spreading) OR (Split ridge expansion) OR (Split crest) (Ridge split) OR (Ridge expansion) OR (Corticotomy) OR (Crestal osteotomy), and other such terms were searched. In addition, manual search for the potential articles was also performed.

### Eligibility criteria and screening process

Articles were included in this systematic review if they met the following inclusion criteria:

1. prospective or retrospective, cohort or case studies/series
2. Cases with 5 or more human subjects.
3. Type of device used for surgery
4. Location of defect
5. Minimum follow up period

References obtained from the search strategy were screened, and duplicates were removed manually, after assessment of title and abstract. This was cross-checked by N.J and G.U.J. A study was included when it met one or more (inclusion) criteria. Only articles written in English were considered for the study.

Number of subjects involved, flap design, implant success rate and gap filling using GBR were analysed on the basis of defect location in maxilla, mandible or both (Tables 1, 2 and 3)[7–43]. Additionally, the various instruments used for ridge expansion were analyzed to focus on the specific type/technique of device used and to identify the most commonly used approach/method. Various characteristics like study type, device specifications, patient discomfort during surgery, ridge width, and complications associated with instrument use were evaluated (Tables 4, 5 and 6) [7–43]. Case series or case reports and clinical studies with missing information were excluded. Articles that mentioned less than 5 subjects and cadaveric/in vitro studies were also excluded.

**Table 1:** Characteristics of included studies (Maxilla).

PUBLICATION	TYPE OF STUDY	NO.OF PATIENTS	FOLLOW UP RATE	FLAP DESIGN FOR SURGERY	TIME OF PROSTHESIS LOADING	SUCCESS RATE OF IMPLANTS	GAP FILLING/ ADDITIONAL GBR ON OUTER BUCCAL REGION
Shaik et al[28]] (2016)	PCS	10	3 mon	-	Prosthesis after 3 mon	-	No
Teng et al[32] (2014)	CR	31	6 mon	full	Implants after 6 mon	-	Gap filling (all cases)
Mounir et al[20] (2014)	RCT	22	1 yr	Full/split	Implants at 6 mon	-	Gap filling (all cases)
Anitua et al[23] (2012)	CS	6	Mean follow up 19 mon	Full	Final Prosthesis loading 1yr after OI.	100%	Gap filling (all cases)
Gonzalez et al[7] (2011)	RCCS	8	24 mon	full	Immediate implant, prosthesis after 4 mon	-	Gap filling (all cases)
Demarosi et al[9] (2009)	PCoS	23	Follow up 3,6,12 mon P.O	full	Immediate implant	97%	No
Ferrigno et al[2] (2005)	PCT	40	6 to 24 mon	partial	Immediate implant insertion	100%	Gap filling (all cases)
Sethi et al[42] (2000)	PCT	150	1,3,6,12 mon after resto	partial	Cemented resto about 8–9 mon after surgery	-	No
Yilmaz et al[43] (1998)	PCoS	16	3 mon	Full/partial	Prosthesis at 6 mon	-	Gap filling (7 cases)

mon = months, OI = osseo-integration, yr = year, resto = restoration, P.O = Postoperatively, RCCS = retrospective case control study, PCT = prospective controlled study, PCoS = prospective cohort study, RCT = randomised control trial, CR = clinical report, PCS = prospective clinical study, CS = case series

**Table 2:** Characteristics of included studies (Mandible).

PUBLICATION	TYPE OF STUDY	NO.OF PATIENTS	FOLLOW UP RATE	FLAP DESIGN FOR SURGERY	TIME OF PROSTHESIS LOADING	SUCCESS RATE OF IMPLANTS	GAP FILLING/ ADDITIONAL GBR ON OUTER BUCCAL REGION
Ella et al[26] (2014)	PCS	32	1 yr	full	Implants after 6 mon	-	Gap filling-53% cases
Rodriguez et Al[33] (2013)	PCoS	143	6 mon after surgery to up to 13 yrs	full	Definite restorations,3–6 mon P.O	-	No
Kawakami et Al[21] (2013)	RCT	12	-	Full	Removable Prosthesis after 6mon	-	Gap filling (all cases)
Scarano et al[34] (2011)	PCoS	22	1 to 6 mon after 1st surgery and sub.	Full	-		Gap filling (all cases)
Sohn et al[15] (2010)	CS	32	-	full	14–17 mon	-	Gap filling (all cases)
Holtzclaw et Al[14] (2010)	CS	13	6–12 mon	full	Implants at 5 mon, resto 4mon after implants	-	Gap filling (all cases)
Enislidis et Al[39] (2006)	PCT	5	1,3,6 mon	partial	Implants after 6 mon	97%	Gap filling (all cases)
Basa et Al[4] (2004)	PCT	21	3–4 mon	Split flap	Immediate implant	-	Gap filling (all cases)
Mazzocco et al[11] (2011)	RCT	8	14,30,90 days	full	Implant at 6 mon	-	No

mon = months, yr = year, P.O = postoperative, resto = restoration, RCCS = retrospective case control study, PCT = prospective controlled study, PCoS = prospective cohort study, RCT = randomised control trial, PCS = prospective clinical study, CS = case series

**Table 3:** Characteristics of included studies (Maxilla & Mandible).

PUBLICATION	TYPE OF STUDY	NO.OF PATIENTS	FOLLOW UP RATE	FLAP DESIGN FOR SURGERY	TIME OF PROSTHESIS LOADING	SUCCESS RATE OF IMPLANTS	GAP FILLING/ADDITIONAL GBR ON OUTER BUCCAL REGION
Crespi et al[10] (2014)	RCT	46	6, 12, 24 mon	Partial	Temporary prosthesis after 3mon	-	No
Demetriades et Al[31] (2011)	CS	15	6 mon	split	Prosthesis after 5mon	97%	Gap filling (all cases)
Anitua et al[22] (2011)	RCoS	15	1, 3, 6, 12 mon, mean follow up 11–28 mon	Full	Abutments placed 3 mon after implant installation	100%	Gap filling (all but 1)
Cortes et al[36] (2010)	CS	21	Min follow up 6 weeks after surgery	-	Prosthesis within 6mon	-	Gap filling (33% cases)
Blus et al[37] (2010)	PCoS	43	3,6,12 mon After loading and then annually	Partial	Prosthesis after 5–6 mon of implant healing	95%-maxilla 100%- mandible	Outer buccal filling (all cases)
Danza et al[18] (2009)	RCS	86	3–35 mon	Full/partial	Final resto within 8 weeks	-	No
Jensen et al[38] (2009)	RCS	40	Followup- 6 mon to 1 yr	Full/partial	Immediate implant insertion	93%	Gap filling (6 cases)
Chiapasco et al[12] (2006)	PCT	45	Mean follow up 20.4 mon	partial	Abutment placed 3–4 mom after surgery	97.3%	No
Laster et al[30] (2005)	CR	9	1 yr	-	Prosthesis after 4 mon	97%	No
Suh et al[40] (2005)	CS	10	2 yrs	Full/partial	Abutments placed at 5–6 mon	100%	No

mon = months, yr = year, resto = restoration, RCoS = retrospective cohort study, PCT = prospective controlled study, PCoS = prospective cohort study, RCT = randomised control trial, CR = clinical report, PCS = prospective clinical study, CS = case series, RCS = retrospective clinical study, Er: YAG = erbium: yttrium- aluminium garnet

**Table 4:** Outcomes based on devices used for surgery (Traditional devices).

PUBLICATION	DEVICE USED FOR BONE EXPANSION	DEVICE SPECIFICATION	COMPANY (BRAND)	WIDTH OF BONE(before and after surgery)	PATIENT DISCOMFORT DURING SURGERY	COMPLICATION (BUCCAL/LINGUAL BONE FRACTURE)
Shaik et al[28] (2016)	Osteotome kit, mallet	-	Sirag surgical Enterprises, Chennai, India	B- 3.94 mm[M] A-7.39 mm[M]	-	2 (buccal)
Crespi et al[10] (2014)	Osteotome, Electrical and hand mallet	-	Sweden Martina, Due Carrare, Padova, Italy) (Magnetic Mallet, Meta-Ergonomica, Turbigo, Milano, Italy	B-2.5 mm[M] A-7.26 mm[M]	1pt-BPPV	-
Teng et al[32] (2014)	Chisel, Mallet, Manual reamers	Reamer size-2, 2.5, 3 mm	Bicon ^®^, Boston, USA	[MI]-2.8 mm tolerable pain and swelling		-
Mounir et al[20] (2014)	Osteotomes	-	-	-	-	-
Kawakami et Al[21] (2013)	Surgical burs, saw, chisel	-	-	B-4 mm	-	-
Gonzalez et al[7] (2011)	Diamond disc/reciprocat ing saw, osteotomes	-	-	B-3.42 mm	-	-
Demetriades et Al[31] (2011)	Osteotomes	-	-	B-3-5 mm	-	1(buccal)
Scarano et al[34] (2011)	Scalpel, chisel, osteotome	-	Bone system, Milano, Italy	B-1,3,5 mm [MI]-3 mm	-	-
Holtzclaw et al[14] (2010)	Chisel	-	-	B-3.72 mm [M] A-7.09mm [M]	-	-
Blus et al[37] (2010)	Osteotomes Conical screws	-	Bone Management System, Meisinger	B-3.3±0.3mm [MI] A- 6 ±0.4mm [MI]	-	-
Jensen et al[38] (2009)	osteotomes	-	-	B-3-4 mm,	-	1-buccal fracture, 1-lingual fracture
Demarosi et al[9] (2009)	osteotomes	Cylindro-conical expansion osteotomes	Straumann ^®^, Germany	B-2.5–4.5 mm A-6-7.5 mm	-	-
Enislidis et al[39] (2006)	Osteotome, Mini blade(chisel)	Ref no 376900	Becton, Dikins on Surgical System, NJ	-	-	-
Ferrigno et al[25] (2005)	Osteotome	Flat with linear tip	GEAS^®^ Impla ntology and Oral Surgery, Udine, Italy	B-3to 5 mm	-	1(Buccal)
Suh et al[40] (2005)	Microsaw Blades scalpel mallet	#15 blade	Friadent, Dentsply	-	-	-
Basa et al[41] (2004)	osteotome	-	-	B-3-4 mm	-	-
Sethi et al[42] (2000)	osteotome	Paraboloid tips	Harley Dental Technical Centre, London, United Kingdom	-	-	-
Yilmaz et al[43] (1998)	Chisel and mallet	-	-	[MI]-2.8 mm	-	-

Pt = patient, BPPV = benign paroxysmal positional vertigo, B = before, A = after, [M] = mean, [MI] = mean increase

**Table 5:** Outcomes based on devices used for surgery (Modern devices).

PUBLICATION	DEVICE USED FOR BONE EXPANSION	DEVICE SPECIFICATION	COMPANY (BRAND)	WIDTH OF BONE(before and after surgery)	PATIENT DISCOMFORT DURING SURGERY	COMPLICATION (BUCCAL /LINGUAL BONE FRACTURE)
Ella et al[26] (2014)	Bone expansion device	2 steel arms with transverse screw	Meisinger	B-3 mm	-	43% cases (buccal)
Rodriguez et Al[33] (2013)	Threaded bone expanders	-	Microdent System, Barcelona, Spain	-	-	1(buccal)
Anitua et al[23] (2012)	Motorized expanders	-	BTI-Ultrasonic, BTI Biotechnolo gy Institute S.L., Vitoria, Spain	B-2.97 mm [M] A-10.3 mm [M]	-	-
Mazzocco et al[11] (2011)	Motorized ridge expander	-	MRE; Biotechnolo gy Institute	B-2-3 mm A-7 mm	-	-
Anitua et al[22] (2011)	Motorized expanders	-	BTI- Ultrasonic^®^, BTI Biotechnolo gy Institute S.L., Vitoria, Spain	B- 4.29 mm[MI] A-7.63 mm[MI]	-	-
Cortes et al[36] (2010)	Motorized bone expanders	Screw assisted bone expanders, ratchet, carrier	Microdent, Barcelona, Spain	B-3-4 mm A-5-6 mm	-	-
Danza et al[18] (2009)	Piezo surgery device	-	Surgibone; Silfradent, Forli, Italy	-	-	-
Chiapasco et al[12] (2006)	Extension crest device	2 surgical steel arms and transverse screw	Extension Crest^®^, Bio srl, Milan, Italy	B- 3–4 mm A-7-8 mm	-	1(Buccal)

B = before, A = after, [MI] = mean increase

**Table 6:** Outcomes based on devices used for surgery (Traditional and modern devices).

PUBLICATION	DEVICE USED FOR BONE EXPANSION	DEVICE SPECIFICATION	COMPANY (BRAND)	WIDTH OF BONE(before and after S urgery)	PATIENT DISCOMFORT DURING SURGERY	COMPLICATION (BUCCAL/LINGUAL BONE FRACTURE)
Sohn et al[15] (2010)	Piezoelectric saw,		SurgyBone, Silfradent, Sofia, Italy Dual Laser;	B-2-4 mm A-not reported	-	5(Buccal)
	Er:YAG laser,	6w,20Hz	Lambda Scientifica, Altavilla Vicentina, Italy			
	Chisel and mallet, osteotome		D. Flanagan, Willimantic, Conn			
Laster et al[30] (2005)	Osteotome, Crest widener	Activation screws	Laster crest widener	[MI]-4-6 mm	-	-

Er: YAG = erbium: yttrium- aluminium–garnet, before, A = after, [MI] = mean increase

### Quality assessment of included studies

#### Risk of bias in included studies

For the RCTs, the quality of trials was determined using the Cochrane Collaboration’s tool [16] for assessing risk of bias. The randomization and allocation methods were designated as adequate, inadequate or not applicable, selective reporting and incomplete/complete outcome data and other bias were designated as yes or no.

1. **Low Risk of Bias**-when all criteria were met (adequate method allocation and positive (yes) response to bias criteria)
2. **Unclear Risk of Bias**-criteria were partly met
3. **High Risk of Bias**-when one or more criteria were not met

For the observational studies, the adapted version of Newcastle-Ottawa [18] (modified) was used. The following topics were evaluated for quality assessment.

1. **Selection** of study groups (sample size calculation, representation of cases included and excluded for ridge expansion, selection of controls [ridge expansion not performed], instrument used [traditional or modern devices for ridge split].
2. **Comparability** of cases and control based on study design, instrumentation used.
3. **Outcome–**follow-up long enough for outcome, success rate of implant, and assessment of results based on whether the bone gap was filled or not. The study was analyzed on the basis of stars given to each parameter. A total of 12 stars were given, out of which studies with 8–12 stars (more than 80% domain fulfilled) were high quality studies, 5–8 were medium quality, and less than 5 were considered low quality studies.

#### Data analysis

The data were collected as tables and pooled according to the characteristics selected. The main criteria decided for the studies were based on the type of devices/instruments used.

## Results and discussion

### Study selection

**Fig 1:**
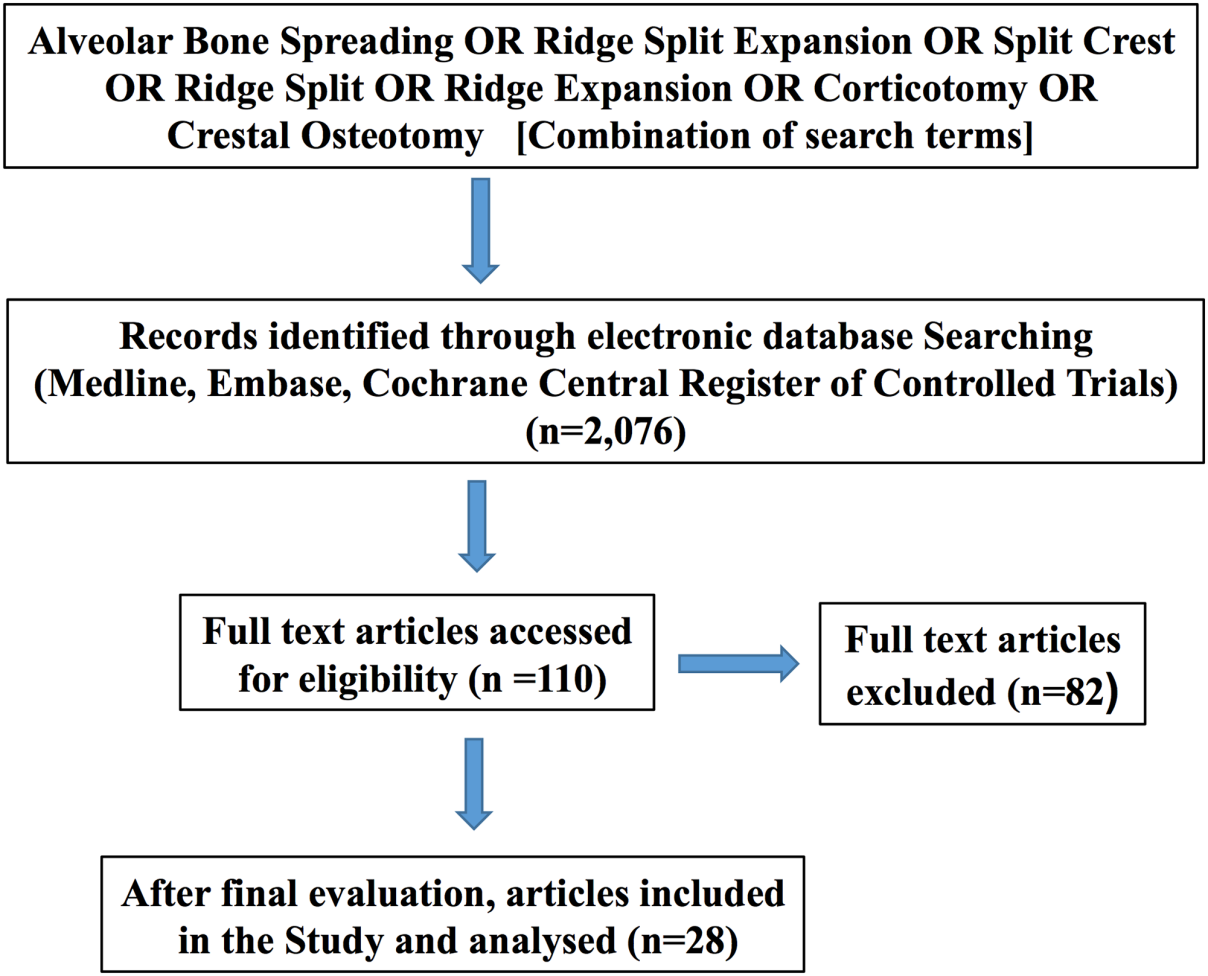
Flow chart of the screening process using different databases.

The search strategy yielded 2,076 articles. Out of these, 2,048 were excluded after review of title or abstract or if they were duplicate articles. After thorough examination of the remaining articles, 28 were found to be potentially fulfilling the inclusion criteria and were subsequently analyzed (Fig 1).

#### Quality of included studies

There were 4 RCTs and the others were observational studies. The quality assessment of the observational studies using modified Newcastle-Ottawa Scale (NOS) is presented in S1 Appendix. According to the NOS, among the studies analyzed, one was of medium quality [19], while all others were of low quality. According to the Cochrane Handbook for Systematic Reviews of Interventions, one RCT [10] was at high risk of bias due to unclear method of allocation and no information on performance, detection, and reporting bias. Other 3 RCTs [11, 20, 21] were at low risk of bias due to 2 having random sequence generation and one study appearing to be free of other sources of bias; these studies reported the expected outcome domains.

### Main result analysis and discussion

After tooth extraction, there are dimensional changes in the alveolar bone, resulting in bone remodelling and reduction in different directions. The bone formation is due to deposition of osteoblasts on the alveolar bone, while osteoclastic activity results in removal/destruction of bone. Most of the alveolar bone lost, is composed of bundle bone. Re fabrication of this portion of alveolar bone is a difficult task but can be accomplished by ridge preservation procedures [12].

The process of ridge split is a vertical osteotomy i.e. cutting of bone downright in the vertical direction to provide space within bone for incorporation of graft material or implants. The alveolar bone is known to be viscoelastic in nature. For very thin alveolar ridges (< 3mm), ridge expansion procedures are very beneficial, as bone in such cases are very soft, have lower elastic modulus, which reinforces their viscoelastic nature and can result in better bone expansion [22].

In this study, we reviewed 4 RCTs and 24 observational studies. The aim of our review was to analyze the various instruments used for the ridge-split procedures and identify the ones used with maximum frequency and high implant success rate. Some of the studies (case series) included in our review did not have control groups, and there was significant heterogeneity of the studies; hence, meta-analysis could not be carried out for our studies.

Among the RCTs, 2 studies demonstrated comparison of the devices used for alveolar ridge expansion. In the first study, by Crespi et al.[10] comparison between electrical and hand mallet was conducted for bone expansion; although no significant differences in results, between the two devices were observed, the electrical mallet was found to be clinically more beneficial than the hand mallet. Bone has different density in different areas and the amount of force applied to the bone (using various instruments) determines the predictability and success of surgical procedure. Use of electrical mallet resulted in low force on the bone with no patient discomfort. The authors [10] reported that the forces were subjected to only to the target areas with minimum trauma to the cranial bones. This may be attributed to the timing of force applied and the movements at the osteotome tips at an energy of 90daN/8μ [10]. In the second study, Mazzocco et al. [11] compared motorized ridge expanders and lateral ridge augmentation for alveolar bone expansion. The differences between the two techniques were statistically insignificant; both were equally effective for successful bone augmentation.

#### Type of instrument used and patient discomfort reported

**Fig 2:**
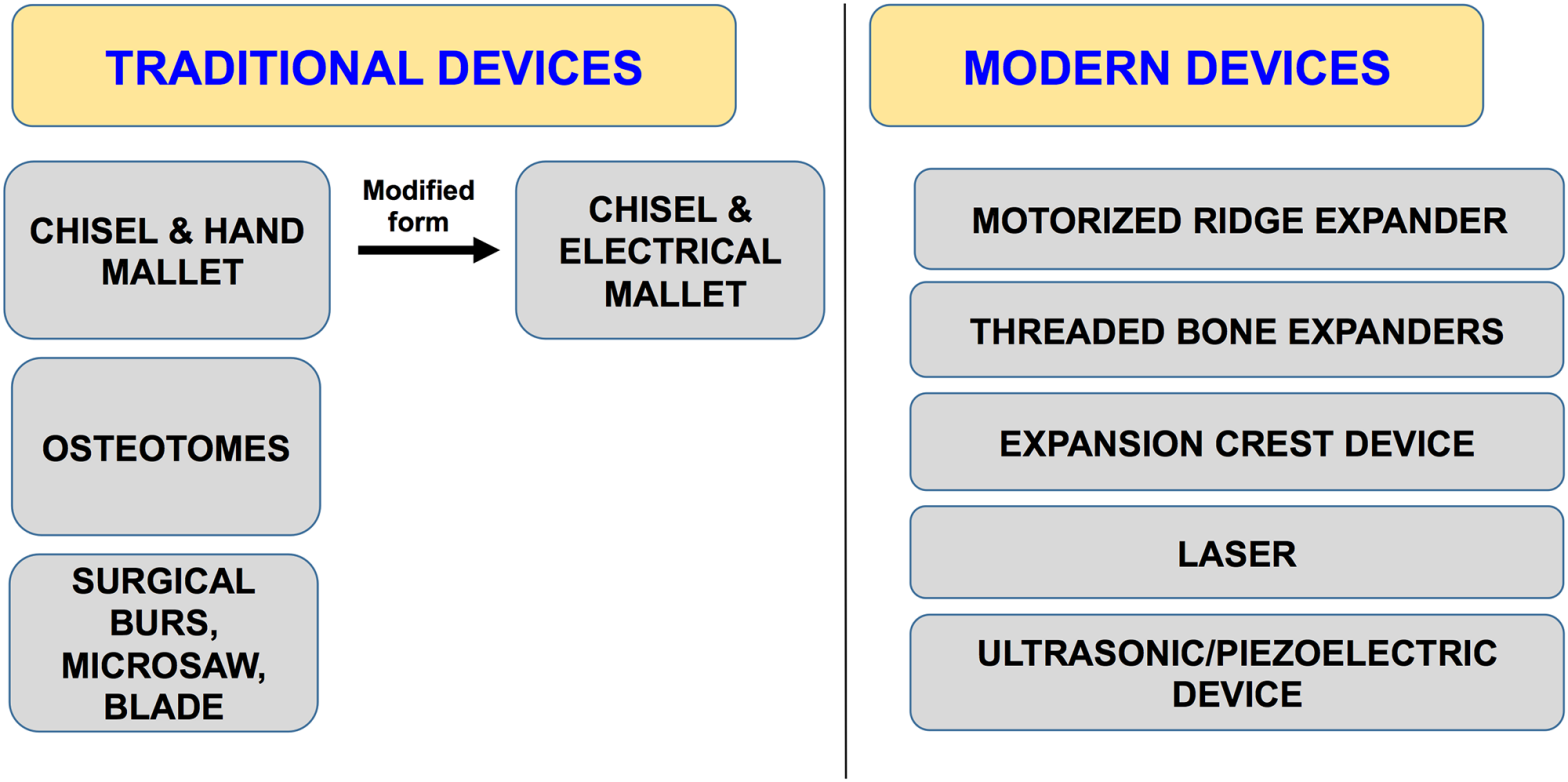
Devices used for ridge expansion.

Ridge expansion can be performed using various kinds of devices (Fig 2). The traditional devices include chisel and mallet; surgical burs; microsaw blades; osteotomes etc. While the modern devices include the motorized bone expanders; expansion crest devices; ultrasonic/piezoelectric devices and bone expanders. With new technologies availability and advancement in the diagnostic field, a shift from the traditional to the modern devices has been seen. The modern devices have an edge over the traditional ones as they act within a short interval of time, cause minimum trauma and prevent bone heating. These factors in turn result in faster bone healing. All this helps to save the clinician’s time and alleviates fear from the patient’s mind as well.

The earliest instruments used for alveolar ridge expansion were chisels and blades. However, using these instruments was difficult as there was no control and precision. This lead to the advent of newer devices for the bone cutting.

We analyzed the type of device used in each study. Some procedures involved use of traditional instrumentation techniques (chisel, blades, osteotomes, mallets, burs and drills), and in some cases, modern devices were used (piezo surgery device, expansion crest device and motorized expander).

**Fig 3:**
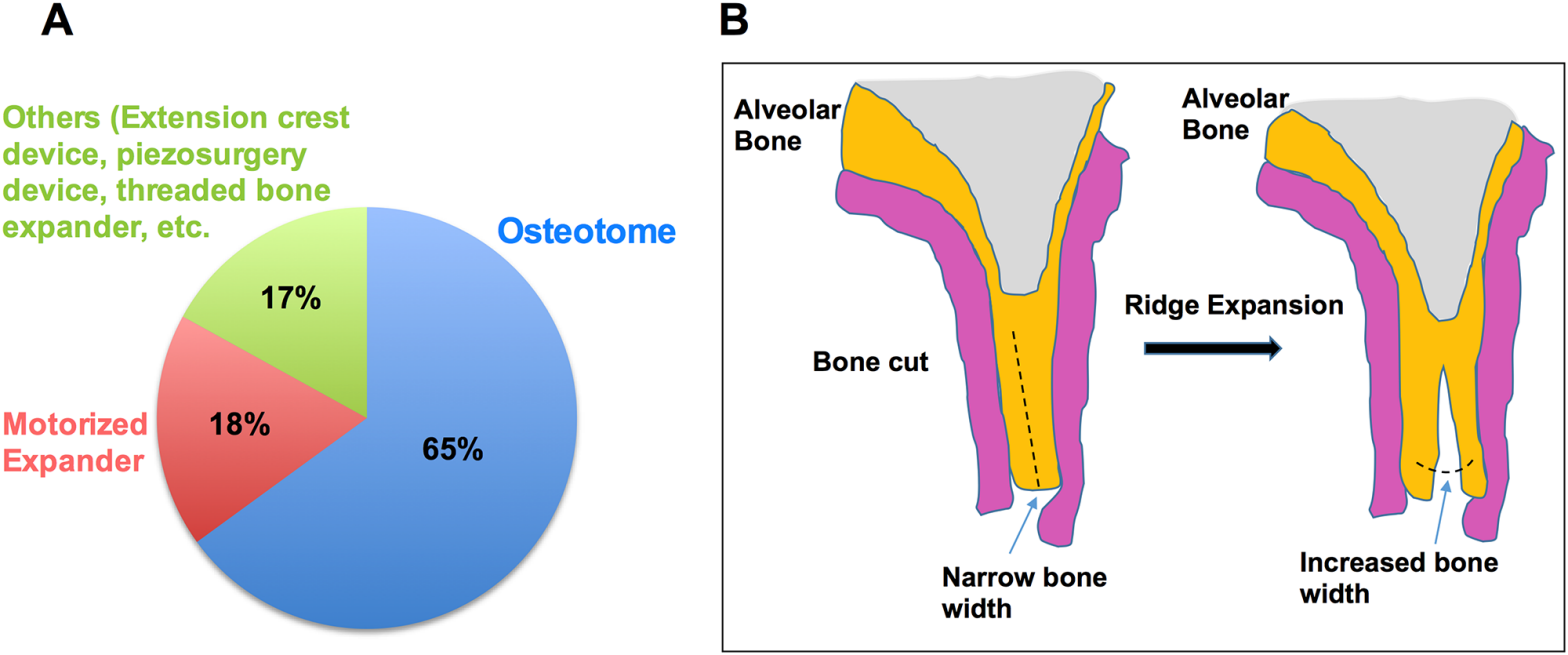
Frequency of the various devices used (A) and schematic representation of ridge split procedure (B).

Of the studies included, 13 reported the use of the traditional device, osteotomes with a frequency of 65%, and amongst the modern expansion devices, there was frequent use of motorized expanders (Fig 3). No significant patient discomfort was observed for any of the included studies, except in one case [10] with vertigo. For all cases, where motorized bone expanders were used, 100% success rate was noted.

#### Bone width

An analysis of the bucco-lingual bone width, before ridge expansion (for the placement of implants) is very important. When the bucco-lingual width is about 3mm, but less than 6mm [4], ridge splitting/ augmentation is recommended. Various instruments are used for the ridge splitting process. In this review, we analysed the initial and final bone width, used for the surgical procedures.

In most of the cases, use of traditional device- the osteotomes was seen very frequently. They showed very good results with an average increase in bone width of about ± 3mm. In the category of the modern devices, piezo surgery devices and motorized expanders were used with an average bone width increase of ± 3.44mm.

Of all the included studies, 8 included data for the alveolar crest initial width (mean = 3.5 mm), while 13 mentioned the final width (mean = 6.65mm); 6 studies mentioned only the difference in alveolar width after expansion (mean = 3.22mm). In cases where motorized bone expanders were used, the mean bone width gain for ridges ≥4 mm was 2.93 mm, while the expansion obtained for ridges <4 mm was 3.95 mm; this indicates that motorized bone expanders provide the best results.

The use of motorized bone expanders has thereby been indicated for extremely resorbed ridges, as they cause minimum tissue damage, facilitate quick and precise movements for the clinician, and can also be placed in apical and occlusal alveolar ridge areas [23] where conventional technique applications are limited.

#### Implant success rate and gap filling

The success rate was variable for all the studies included, with an average of 97%, irrespective of whether gap filling with a barrier membrane was done or not. For all the studies, which reported the success rate of implants, osteotomes were used. Anitua et al. [22, 23] also reported 100% success rates using motorized expanders and piezoelectric device; however, these devices are still not used commonly.

The gap filling for ridge expansion procedures may be done using collagen or mineral graft material. The inter-positional gap filling and the outer buccal region filling (after GBR) frequency was analyzed, in this study. In fourteen studies, gap filling for all subjects was done, while in 4 cases selective gap filling was done. Gap filling may or may not influence the final outcome of implant success [24, 25] however, since the graft material takes part in the bone remodelling process, it expedites the healing process. Ella et al. [26] advocated the use of bone filling substitutes, especially in the horizontally expanded sites as it resulted in reduced resorption around the implant bony walls. The direct contact between the bone walls and implant is reduced with bone substitutes acting as a cushion against ischaemic resorption with some gain in bone volume. Jensen et al. [27] have reported that generally gap filling of less than 3mm do not require any graft material except collagen sponge. However, the amount of gap width which necessarily requires any grafting is difficult to determine and whether grafting facilitates or impedes osseo integration remains uncertain.

#### Complications due to devices used

The most common complications observed during, or on completion of the ridge expansion procedure, was bone fracture. The mandibular bone has thicker cortical plate and is less flexible than the maxilla, hence the rate of bone fracture during ridge expansion (especially in the buccal region) is more for mandibular region. Studies have shown that ridge expansion with osteotomes or implant insertion [25] may lead to fracture of the cortical plate (mostly labial). Of all the studies included, 7 reported bone fracture, with buccal fracture being more common. In a study by Ella et al. [26] buccal bone fracture occurred because expansion was done in a narrow ridge (width, 3mm). Shaik et al. [28] reported fracture of the buccal plate due to pulling of the osteotome (after tapping), more in the buccal direction. To prevent bone fracture, Hotzclaw et al. [14] used a modified technique whereby apical hinge cuts were used, which were not fully in the buccal plate so that some mobilization of the buccal plate could be achieved. It was observed that buccal bone fracture was frequent with osteotome usage, and use of motorized expanders was associated with no reported bone fracture or any other complications.

### Comparison with other studies / reviews

Till now three main systematic reviews [5, 24, 29] have been carried out, which study the survival rate of titanium implants after ridge expansion procedure, assessment of predictability, dimensional change, and other factors associated with ridge expansion and evaluation of clinical and radiological analysis of ridge splitting with or without GBR. In comparison to prior systematic studies, this analysis did not include any animal studies. We aimed to analyze the type of instruments used for the ridge expansion procedure and found that osteotomes were the most frequently used for such procedures.

This review shows that osteotomes remain the most popular device, especially in the maxillary bone [9] due to the lower possibility of heat generation and the increased initial stability because of lateral condensation of bone [7]. The osteotomes do not surgically remove the bone during preparation, rather they exert pressure in the form of lateral compression facilitating increased bone density and successful primary retention of dental implants. Further, there is less risk of crestal bone loss around the implant, and hence, less fear and anxiety related to implant failure [30]. However, there are several disadvantages to this technique. It cannot achieve vertical bone height, and only width gain is possible. Ridge split surgeries using osteotomes may be difficult to perform and require a lot of skill; there is considerable operator dependency involved as well [6, 31]. Amongst the modern expansion devices, the motorized bone expanders and piezoelectric surgery devices have shown promising results. The conventional osteotomy techniques [44] cannot always prevent trauma to the nerves and blood vessels.

Piezosurgery is a type of ultrasonic instrumentation. Piezoelectric bone surgery or piezosurgery or ultrasonic osteotomy is a procedure in which bone cutting is done using low frequency ultrasonic vibrations. The concept of ultrasonic osteotomy/piezo-surgery was introduced which is based on the reciprocal piezo effect [45]. A polarized piezo-ceramic receives a certain amount of voltage which causes deformation of piezoelectric crystals; creating alternate expansion and contraction of the material. This helps in selective cutting of bone without any damage to the soft tissue and other surrounding structures. It appears that the expander works not only for its intended purpose, but also as a condenser of the trabecular bone [11]. Piezo-surgery has also been shown to be feasible in inferior alveolar nerve surgery as it favors smaller osteotomies and preserves the neurovascular bundle without any nerve injury. Additionally, it is known to reduce dental fear and patient (psychological) stress and has very less noise generation [15]. The motorized expanders and ultrasonic surgery system are easier to use, provide more alveolar bone width gain in comparison to the traditional devices, and cause less trauma to the bone compared to the traditional/conventional instruments such as mallets and osteotomes. However, their cost is a limiting factor, and therefore, osteotomes remain a popular mode of instrumentation for ridge expansion procedures.

### Limitations

In this review, most of the current included studies, were of low quality and had limited scientific evidence. Also, most studies included were case series with methodologies representing low levels of evidence. The literature study was confined to English publications, which may have introduced a selection bias. Additional studies that provide a successful comparison of the devices used for ridge width expansion, need to be performed. For a better determination of the most favorable ridge expansion technique [5], well designed studies according to CONSORT guidelines [46] may be needed.

## Conclusion

Based on the results from the available studies, it was found that the successful use of alveolar ridge expansion device is dependent on several factors. Patient discomfort during surgery, the gap filling with GBR, before or after surgery and complications seen during or after surgery are possible factors that affect the success outcome of the ridge expansion devices.

The osteotomes are the most widely used conservative devices for ridge expansion due to their ease of usage and availability. Using an osteotome allows excellent (manual) control with adequate determination of the implant axis. The device is simple to use and very cost effective, hence can be used on a large scale. However, piezoelectric device and other modern devices are being increasingly used as new devices for crest ridge expansion. They are more suitable to prevent any trauma to the vulnerable structures like mucosa, nerves and blood vessels. Since there is less trauma to the bone, it results in faster healing. These devices should be used more in the future.

## Supporting information

S1 Appendix Quality assessment of the observational studies (NOS). (DOCX)

S2 Appendix PRISMA checklist. (DOCX)
