# Supplementary material for: Types of devices used in ridge split procedure for alveolar bone expansion: A systematic review
Source: PLoS One. 2017 Jul 21;12(7):e0180342. doi: 10.1371/journal.pone.0180342 (PMC5521746; doi:10.1371/journal.pone.0180342)
Supplement: S1 Appendix — (DOCX) [file pone.0180342.s001.docx]

**S1 Appendix. QUALITY ASSESSMENT OF THE OBSERVATIONAL STUDIES**

**(NOS)**

1. Sample size was not given for any study.
2. Follow up was adequate in 9 cases for the outcome analysis.
3. Ascertainment of exposure using surgical records was available for 4 cases.
4. Device comparability for the study designs was available for only 2 cases.
5. Demonstration of outcome at the start of the study was seen in 3 cases.
6. 14 cases showed clear inclusion and exclusion criteria.
7. Only one study defined the number of patients as the unit of analysis.
8. The success rate of implants was available for 11 cases.
9. Alveolar ridge atrophy was not adequately reported except in 2 cases.
10. Selection of controls was adequately reported for only 2 cases
11. None of the studies described blinded assessment of split crest outcomes
12. Statistical analysis was given for 8 cases.
